# Functional, Patient‐Centered and Bone Remodeling Outcomes in Two Narrow‐Diameter Implant Retained Mandibular Overdenture Wearers After 5 Years of Follow‐Up: A Prospective Case Series

**DOI:** 10.1111/clr.14471

**Published:** 2025-07-11

**Authors:** Anna Paula Da Rosa Possebon, Fernando Antônio Vargas Júnior, Fernanda Isabel Román Ramos, Otacílio Luiz Chagas‐Júnior, Luciana de Rezende Pinto, Fernanda Faot

**Affiliations:** ^1^ Universidade Federal de Pelotas (UFPel) Pelotas Rio Grande do Sul Brazil; ^2^ Graduate Program in Dentistry Universidade Federal de Pelotas (UFPel) Pelotas Rio Grande do Sul Brazil

**Keywords:** mandibular bone remodeling, mandibular overdentures, masticatory function, oral health‐related quality of life

## Abstract

**Objectives:**

This prospective longitudinal case series investigated changes in functional, radiographic, and patient‐centered outcomes in users of two narrow‐diameter implant‐retained mandibular overdentures (IMO) over a 5‐year follow‐up.

**Materials and Methods:**

Twenty‐four patients from a previous 3‐year study (8 man, 16 women, 71.58 ± 7.03 years, mean mandibular edentulism duration 23.9 ± 14.4 years) were invited for a 5‐year follow‐up evaluation of radiographic and functional parameters. Masticatory function (MF) was assessed using the Swallowing Threshold Test (ST), with X50 and B cut‐off values indicating satisfactory performance. Oral health‐related quality of life (OHRQoL) was evaluated using the OHIP‐Edent questionnaire. The Posterior Mandibular Area Index (PAI) was analyzed using digital panoramic radiographs. Data was analyzed using multilevel mixed‐effects linear regression.

**Results:**

Twenty participants completed the 5‐year follow‐up evaluation. ST_X50 (4.00–3.70; *p* ≤ 0.01; 95% CI: 0.47–1.82) reached a significant reduction along with an increased number of masticatory cycles (59.33–65.60; *p* ≤ 0.01; 95% CI: 0.32–1.11) between years. The number of IMO wearers presenting satisfactory mastication increased but persisted as unsatisfactory in nine participants by year 5. Declines in the Functional Limitation and Physical Disability domains of OHRQoL were noted, with unsatisfactory mastication correlating with higher pain (coef: 2.5; *p*: 0.03; 95% CI: 0.25–4.74) and psychological disability scores (coef: 0.94; *p* ≤ 0.01; 95% CI: 0.19–1.68). PAI increased significantly (1.13–1.68; *p* ≤ 0.01; 95% CI: 0.24–0.56), indicating ongoing bone remodeling.

**Conclusions:**

Although MF and bone parameters improved over 5 years, persistent unsatisfactory mastication continued to negatively affect quality of life. These findings underscore the interdependence between functional and patient‐centered outcomes in IMO users.

**Trial Registration:**

The Brazilian Registry of Clinical Trials (ReBEC), Trial identification: UTN code: U1111‐1259‐4127

## 1 Introduction

The literature consistently supports that the minimum recommended rehabilitation for completely edentulous patients involves the use of mandibular overdentures retained by two implants (IMO) (Feine et al. 2002; Thomason et al. 2012). Most existing studies report outcomes from short‐ and medium‐term follow‐ups (Fueki et al. 2007; Al‐Omiri and Karasneh 2010; Boven et al. 2015; Schuster et al. 2017; Marcello‐Machado, Faot, Schuster, Bielemann, Chagas Júnior, et al. 2018; Possebon et al. 2018; Iwaki et al. 2019), leaving gaps in the understanding of IMO performance over extended periods, as well as their influence on masticatory function (MF), mandibular ridge resorption, and oral health‐related quality of life (OHRQoL).

The biomechanical stability provided by implants over time is generally believed to contribute to the preservation of mandibular bone, particularly in the posterior region, thereby supporting the long‐term success of IMO rehabilitation. Several studies have investigated the changes in the posterior mandibular ridge following IMO use (Tweed 1969; Jacobs et al. 1992; Suenaga et al. 1997; Burns 2000; Wright et al. 2002; Kordatzis et al. 2003; de Jong et al. 2010; Elsyad et al. 2017; Oh et al. 2020; Possebon et al. 2020). Although earlier studies reported continued posterior ridge resorption in IMO users (Suenaga et al. 1997; Burns 2000; Kordatzis et al. 2003), more recent findings suggest that posterior bone loss may be reversible over a 3‐year follow‐up period (Possebon et al. 2020; Faot et al. 2025). Similarly, short‐term bone gains ranging from 1.6% (Wright et al. 2002) to 17% (Reddy et al. 2002) have been observed, particularly among individuals with initially low bone availability (Reddy et al. 2002; Marcello‐Machado et al. 2017; Possebon et al. 2020). However, the overall evidence remains inconclusive. A retrospective radiographic study by Kremer et al. (2016), with a mean follow‐up of 11 ± 4.75 years, monitored bone resorption at three distinct mandibular sites in IMO users and observed region‐specific resorption patterns, with significant bone loss persisting mainly in the posterior region of the mandible, particularly near the distal edge of the denture flange, with median annual resorption rates of 0.15 mm (left) and 0.18 mm (right). These divergent findings highlight the complexity of bone remodeling under functional loading and suggest that the long‐term preservation of the posterior mandibular ridge remains uncertain, warranting further investigation.

Functional improvements associated with the use of IMOs can be assessed through both objective and subjective measures. Objectively, the progression or decline of masticatory function (MF) is evaluated, while subjectively, patient self‐reports are translated into oral health‐related quality of life (OHRQoL) indicators. Monitoring both outcomes over time is essential to ensure sustained patient satisfaction with treatment. Consequently, long‐term follow‐up of this rehabilitation approach is critical to maintaining the initial functional benefits observed in the early years (Al‐Omiri and Karasneh 2010; Schuster et al. 2017; Enkling et al. 2017; Possebon et al. 2018), which may diminish due to insufficient understanding of the long‐term performance of IMOs. From a functional standpoint, masticatory performance is a well‐documented topic in the literature, with reported improvements in chewing efficiency, food bolus homogenization, and the number and duration of masticatory cycles during the initial years of IMO use (Fontijn‐Tekamp et al. 2004; Stellingsma et al. 2005; van der Bilt et al. 2010; van der Bilt 2011; Marcello‐Machado, Faot, Schuster, Bielemann, Nascimento, et al. 2018; Possebon et al. 2018, 2020). Regarding patient self‐perception, studies have shown a significant enhancement in OHRQoL within the first 3 months of IMO use (Miranda et al. 2019), especially by addressing functional problems such as mandibular denture retention issues. These improvements tend to remain stable for up to 5 years (Matthys et al. 2019). However, it is important to acknowledge that OHRQoL can be influenced by ongoing maintenance and prosthetic interventions. Frequent maintenance needs can negatively impact long‐term OHRQoL over time, emphasizing the need to manage these factors to ensure long‐term patient satisfaction (Zhang et al. 2019; Matthys et al. 2019; Canallatos et al. 2020; Schuster et al. 2022).

Few studies have evaluated the clinical and functional performance of IMOs beyond 2 years (Al‐Nawas et al. 2012; Zweers et al. 2015; Quirynen et al. 2015; Müller et al. 2015; Schuster et al. 2018, 2019; Possebon et al. 2020, 2021). The long‐term follow‐up of completely edentulous patients—particularly those rehabilitated with IMOs—is essential to ensuring the success and durability of prosthetic treatment, a point reinforced by previous studies (Trullenque‐Eriksson and Guisado‐Moya 2014; Balaguer et al. 2015) which highlight the importance of longitudinal monitoring by demonstrating high implant survival rates over extended periods. For IMO‐rehabilitated patients, regular maintenance is particularly important due to the direct connection between the prostheses and implants and the overall condition of the mandibular ridge. Therefore, long‐term monitoring not only ensures prosthesis stability but also facilitates early detection of potential complications or the need for adjustments. In this context, the present study aimed to investigate changes in functional, radiographic, and patient‐centered outcomes among IMO users over a 5‐year follow‐up period. The null hypothesis tested was that there would be no significant changes in any of the assessed outcome variables during this time.

## 2 Methodology

### 2.1 Study Design

This prospective longitudinal case series presents the results of a 5‐year follow‐up of completely edentulous patients rehabilitated with mandibular overdentures supported by two narrow‐diameter implants (2.9 × 10 mm; Facility‐Equator System, Neodent, Brazil). The study was approved by the Research Ethics Committee of the Faculty of Dentistry, Federal University of Pelotas (CAAE: 47353215.4.0000.5318; Report number: 3.725.829), conducted in accordance with the 2008 Declaration of Helsinki, and adhered to the Strengthening the Reporting of Observational Studies in Epidemiology (STROBE) guidelines (Bastuji‐Garin et al. 2013). The sample size was recalculated based on differences in masticatory function reported by van der Bilt et al. (2010), using the difference in means of chewed particle size between two periods (Period 1: mean = 3.7 mm, SD = 0.8; Period 2: mean = 2.4 mm, SD = 0.4). With 80% power, *α* = 5%, and a 20% allowance for potential dropouts, 10 participants were needed to detect significant differences in masticatory function. Therefore, the 24 patients from a previous 3‐year study (Possebon et al. 2020) were invited to return for evaluation of radiographic and functional outcomes after 5 years of IMO use. All participants provided a written informed consent form.

Patients were selected from those receiving treatment at the Complete Dentures Clinic at the Federal University of Pelotas. Inclusion criteria included difficulties with retention and stability of mandibular dentures and complaints of impaired mastication. The initial sample consisted of 24 individuals, most of whom were female (66.7%) and self‐identified as white (91.7%). Regarding marital status, 50.0% were married, 20.8% widowed, 16.7% single, and 12.5% divorced. Low educational attainment predominated, with 66.7% not completing primary education. Most participants (79.2%) reported a monthly income below one minimum wage. The mean duration of edentulism was 31.04 years (SD = 13.04) in the maxilla and 23.95 years (SD = 14.39) in the mandible, with 41.7% edentulous in the mandible for over 25 years. Mandibular bone atrophy was present in 54.17% of the sample. Regarding the anteroposterior relationship, 50.0% were classified as Class III. Facial patterns were relatively evenly distributed among dolichofacial (37.5%), brachyfacial (33.3%), and mesofacial (29.2%) types. Reported comorbidities included hypertension (62.5%), arthritis (50.0%), diabetes mellitus (25.0%), and psychiatric disorders (20.8%). Only 12.5% of participants were smokers.

All participants received new conventional complete dentures fabricated using heat‐polymerized acrylic resin (VIPICRIL Plus; VIPI, Pirassununga, Brazil) and acrylic resin teeth, arranged in bilateral balanced occlusion. Following a 3‐month adaptation period, two narrow‐diameter implants (2.9 mm, Facility‐Equator System, Ti grade V, NeoPoros surface; Neodent, Curitiba, Brazil) were placed in the interforaminal region by a single experienced surgeon (OLCJ). Healing abutments were installed immediately. After a 3‐month osseointegration period, stud‐type attachments (Equator System) were placed, and the mandibular overdentures were loaded.

### 2.2 Masticatory Function: Functional Outcome

Masticatory function was evaluated using the swallowing threshold test (ST), in which participants chewed a standardized portion (3.7 g) of artificial test food (Optocal) until the natural urge to swallow occurred. A trained and calibrated examiner (APDRP) manually recorded the number of masticatory cycles, each defined as a complete mandibular opening and closing. Total chewing time was recorded with a digital stopwatch to assess both masticatory duration and rhythm. After chewing, the material was expelled onto filter paper, dried at room temperature for 7 days, and subjected to sieve fractionation using a shaker and a stack of sieves with mesh sizes from 5.6 to 0.5 mm. The retained material was weighed, and the ST_X50 (mean particle size) and ST_B (particle homogeneity) values were calculated using the Rosin‐Rammler formula. ST_X50 represents the theoretical sieve opening through which 50% of chewed particles pass, while ST_B reflects the homogeneity of particle size. To complement the analysis, masticatory efficiency (ME_5.6 and ME_2.8), defined as the percentage of material retained on sieves with openings of 5.6 and 2.8 mm, respectively, along with the number and duration of masticatory cycles, were also recorded (Fontijn‐Tekamp et al. 2000). Patients were then categorized into groups based on satisfactory or unsatisfactory masticatory function using a cutoff of 3.68 for ST_X50 (Witter et al. 2013) and the median value of 2.61 for ST_B (Possebon et al. 2018). Values above the cutoff were considered unsatisfactory and values below, satisfactory.

### 2.3 Posterior Mandibular Area Index: Radiographic Outcome

**FIGURE 1:**
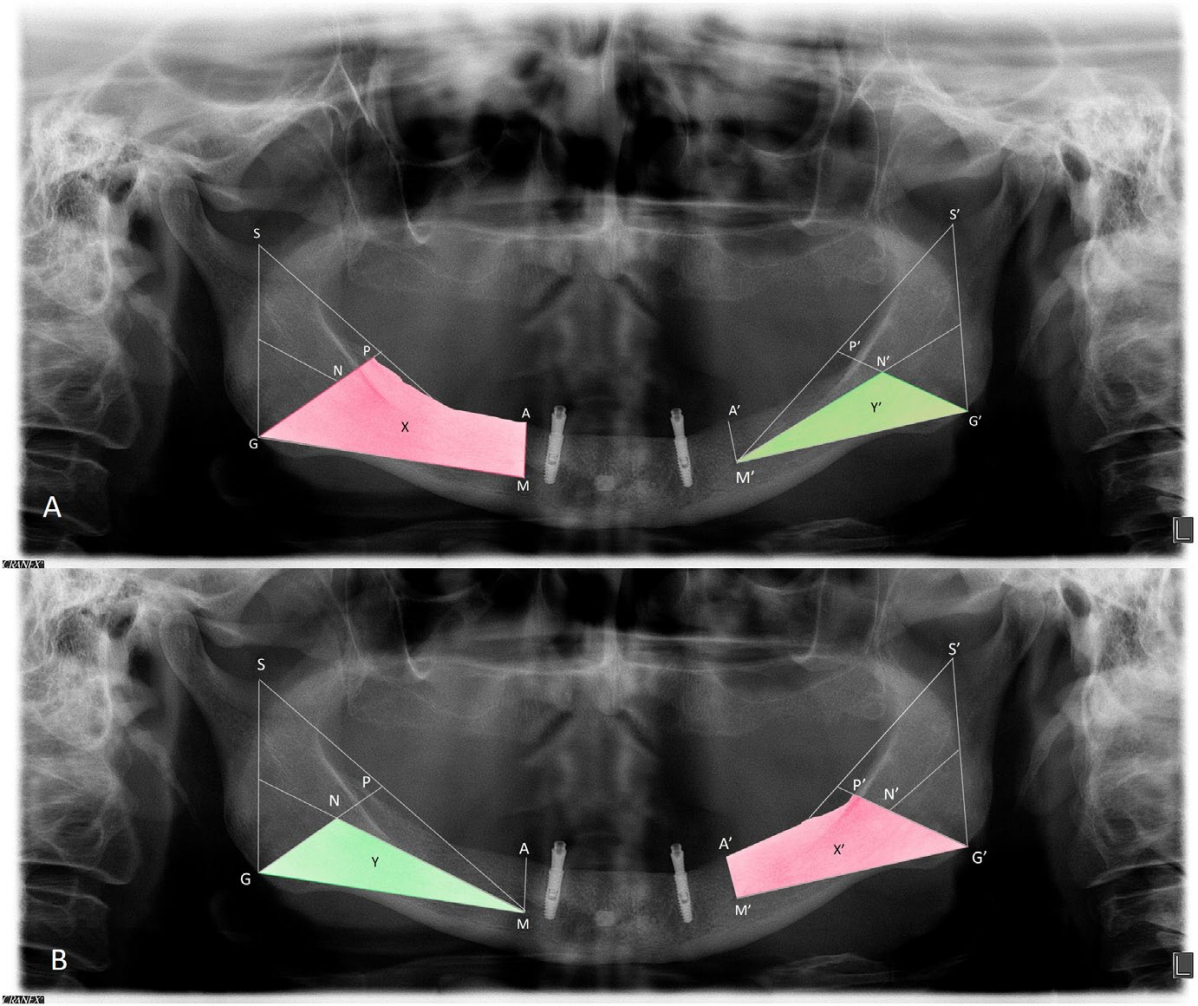
Demonstration of the areas for calculating the PAI in the mandible using panoramic radiographs to determine experimental and reference area regions: (A) right reference area (Y) and left experimental area (X); (B) right experimental area (X′) and left reference area (Y′).

The Posterior Mandibular Area Index (PAI) was assessed using methods described by Wright et al. (2002), Kordatzis et al. (2003), and Elsyad et al. (2017). Panoramic radiographs were obtained using a DentaScan device (12.7 × 30 cm; Dürr Dental, Germany) and digital phosphor plate sensors. Images were processed and adjusted for brightness and contrast using DBSWIN software. Proportional measurements were used to minimize magnification and distortion errors. The reference points and lines were drawn using Photoshop, and areas were delineated bilaterally using ImageJ software. The experimental area was defined by the line connecting the gonion (G/G′) to the lower margin of the mental foramen (M/M′) and the residual ridge crest. The reference area comprised a triangle formed by G/G′, M/M′, and a point N/N′ located at the center of the triangle G/G′–M/M′–sigmoid notch (S/S′), a region unaffected by ridge resorption. The boundaries of the experimental area were demarcated using lines MG and M′–G′, AL and A′–L′ (from crest to lower mandibular edge, perpendicular to MG and M′–G′), MN and M′–N′, and GP and G′–P′, with GN and G′–N′ extended to meet the crest at P and P′. The PAI was calculated as the ratio between the experimental and reference areas, and the mean of each side was reported (Figure 1). Changes in PAI over time quantified ridge resorption. All measurements were performed in duplicate by the same examiner (FIRR), with a 1‐month interval. Intraclass correlation coefficients (ICC) were calculated for reliability.

### 2.4 OHIP‐Edent: Patient‐Centered Outcome

Patient‐centered outcomes were measured using the OHIP‐Edent questionnaire, which includes seven domains and a global score. Responses were assigned as numerical values: 0—never, 1—sometimes, and 2—almost always (Souza et al. 2007). Higher scores in each domain indicate worse OHRQoL (Possebon et al. 2020). The effect size (ES) was calculated based on the final OHIP‐Edent scores and classified as small (0.2 ≤ ES < 0.5), moderate (0.5 ≤ ES < 0.8), or large (ES ≥ 0.8).

### 2.5 Statistical Analysis

A multilevel linear mixed‐effects regression analysis was conducted to account for the hierarchical structure of the data. This approach enabled the evaluation of trends in variable changes by incorporating a random intercept, facilitating the modeling of individual change trajectories based on repeated measures. Time points were treated as fixed effects to assess linear trends, while participants' age was included as a random effect. The fifth year was designated as the reference point for comparisons, with data from the third and first years incorporated to assess longitudinal changes. Additionally, the association between masticatory function (categorized as satisfactory or unsatisfactory) and OHRQoL was analyzed using multivariate regression analysis. All variables were retained in the regression models regardless of statistical significance. Statistical analyses were performed using STATA SE 14.1 software (StataCorp, College Station, TX, USA), with statistical significance set at *p* ≤ 0.05.

## 3 Results

A total of 20 patients completed the 5‐year follow‐up evaluation. Four participants dropped out during the fourth year: two due to death and two due to loss of contact during the COVID‐19 pandemic. Table 1 presents the retention and dropout flow from the first year of follow‐up of IMO users. The final sample consisted of 14 women and 6 men, with a mean age of 71.5 years (±7.03) years. The intraclass correlation coefficient for the PAI analysis was 0.99. Regarding changes in the posterior mandibular region, the PAI increased from 1.13 to 1.68 between years 1 and 5 (*p* ≤ 0.01; 95% CI: 0.24–0.56) and from 1.15 to 1.68 between years 3 and 5 (*p* ≤ 0.01; 95% CI: 0.20–0.57).

**TABLE 1:** Retention vs. dropout flow based on the first year of IMO follow‐up, *n* = 26 participants.

Year	Retained	Lost this period	Retention %	Dropout %
1	26	0	100	0
2	24	2 (1 death, 1 relocation)	92.3	7.7
3	24	0	92.3	0
4	20	4 (2 deaths, 2 lost to COVID‐19)	76.9	15.4
5	20	0	76.9	0

**FIGURE 2:**
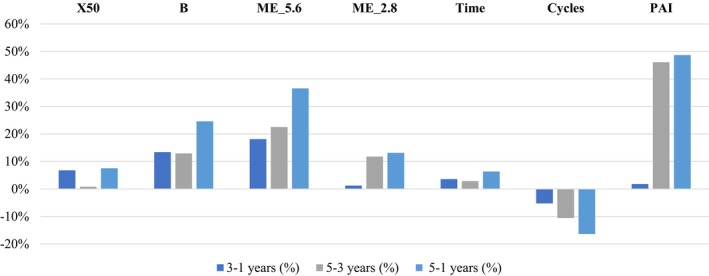
Percentage of gradual changes in the outcomes for 5 years.

Analysis of functional variables (Tables 2 and 3; Figure 2) over time revealed a significant change in ST_X50 between 1 and 5 years (*p* ≤ 0.01; 95% CI: 0.47–1.82), with a decrease in the mean value from 4.00 to 3.70. As for ST_B values, which reflect particle homogeneity, no significant changes were observed over the evaluated period. Significant changes were detected in masticatory cycles. The number of cycles increased from 59.33 to 65.60 between years 3 and 5 (*p* ≤ 0.01; 95% CI: 0.32–1.11). Additionally, the duration of masticatory cycles showed a reduction from 54.56 to 53.01 s between years 3 and 5 (*p* ≤ 0.01; 95% CI: 0.38–0.93), and a reduction from 56.59 to 53.01 s between years 1 and 5 (*p*: 0.03; 95% CI: 0.03–1.09). With regard to the amount of material retained on each sieve, a significant reduction was observed in sieve ME_5.6 across both periods (1–5 years: *p* ≤ 0.01; 95% CI: 0.33–1.87 and 3–5 years: *p* ≤ 0.01; 95% CI: 0.47–1.77), with retention decreasing from 22.9% to 14.53% in the first period, and from 18.75% to 14.53% in the latter 2 years. For sieve ME_2.8, a significant increase in retention was observed, from 23.28% to 26.02% between years 3 and 5 (*p* ≤ 0.01; 95% CI: 0.23–1.42). In years 1 and 3, 12 and 9 individuals, respectively, exhibited unsatisfactory mastication. In the fifth year, nine individuals demonstrated unsatisfactory ST_X50 and 10 exhibited unsatisfactory B.

**TABLE 2:** Means (SD, standard deviations), minimum and maximum values for masticatory function and radiographic parameters over 5‐year follow‐ups.

	1 Year	3 Years	5 Years
	Mean (SD) [min–max]	Mean (SD) [min–max]	Mean (SD) [min–max]
**Masticatory function**			
ST_X50	4.00 (1.32) [2.24–6.57]	3.73 (1.21) [1.76–6.24]	3.70 (0.65) [2.60–5.45]
ST_B	3.66 (2.06) [2.26–10.20]	3.17 (1.29) [2.06–6.91]	2.76 (0.66) [2.12–5.28]
Cycles	56.37 (22.57) [37–134]	59.33 (23.31) [33–117]	65.6 (21.42) [31–130]
Time	56.59 (27.50) [23.49–145.30]	54.56 (18.36) [29.37–102.35]	53.01 (21.71) [0.39–93.74]
ME_5.6	22.9 (25.92) [0–85.20]	18.75 (20.83) [0–71.24]	14.53 (11.81) [0–44.07]
ME_2.8	23.0 (11.34) [1.62–52.33]	23.28 (12.03) [0–42.02]	26.02 (7.25) [7.85–37.42]
**Radiographic**			
PAI	1.13 (0.21) [0.75–1.62]	1.15 (0.22) [0.83–1.63]	1.68 (0.37) [1.06–2.63]

**TABLE 3:** Multilevel mixed‐effects regression for functional and radiographic variables.

	5 Years (Ref)	1 Year		3 Years	
	Coef. [95% CI]	Coef. [95% CI]	*p*	Coef. [95% CI]	*p*
ST_X50	1.00	**1.14** [0.47:1.82]	**0.01**	0.58 [−0.14:1.32]	0.11
ST_B	1.00	−0.48 [−1.55:0.59]	0.37	−0.16 [−1.10:0.73]	0.71
Time	1.00	**0.56** [0.03:1.09]	**0.03**	**0.65** [0.38:0.93]	**0.00**.
Cycles	1.00	0.13 [−0.34:0.60]	0.59	**0.72** [0.32:1.11]	**0.00**
ME_5.6	1.00	**1.10** [0.33:1.87]	**0.00**	**1.12** [0.47:1.77]	**0.00**
ME_2.8	1.00	0.36 [−0.29:1.03]	0.27	**0.83** [0.23:1.42]	**0.00**
PAI	1.00	**0.40** [0.24:0.56]	**0.00**	**0.38** [0.20:0.57]	**0.00**

*Note:* Values in bold represent statistically significant differences (*p* ≤ 0.05).

Regarding outcomes from the OHIP‐Edent instrument (Table 4), negative changes were identified in some domains such as Functional Limitation (*p* ≤ 0.01; 95% CI: 0.20–1.44), Physical Disability (*p* ≤ 0.01; 95% CI: 0.31–1.86), and Global score (*p* ≤ 0.01; 95% CI: 0.16–1.83) when comparing the fifth and first year. In contrast, a slight improvement was observed in the Handicap domain during the same period (*p*: 0.03; 95% CI: 0.01–0.39). The domains showing large effect sizes between the fifth and first years were (Table 5): Functional Limitation (ES = 2.7), Psychological Discomfort (ES = 1.0) and Pain (ES = 0.8), which maintained the same effect sizes when comparing the fifth and third years. Regarding the impact of masticatory performance on quality of life (Table 6), unsatisfactory mastication (as indicated by ST_X50) was associated with negative predictors of OHRQoL in the fifth year. Specifically, unsatisfactory mastication significantly impacted pain outcomes (coef: 2.5, *p*: 0.03; 95% CI: 0.25–4.74) and psychological disability (coef: 0.92; *p*: 0.01; 95% CI: 0.19–1.68).

**TABLE 4:** Multilevel mixed‐effects regression for the OHIP‐Edent instrument.

OHIP‐Edent domains	5 Years (Ref)	1 Year		3 Years	
	Coef. [95% CI]	Coef. [95% CI]	*p*	Coef. [95% CI]	*p*
Functional Limitation	1.00	**0.82** [**0.20**:**1.44**]	**0.01**	−0.52 [−1.05:0.01]	0.06
Pain	1.00	0.75 [−0.25:1.76]	0.14	−0.06 [−0.87:0.74]	0.87
Psychological Discomfort	1.00	−1.39 [−3.23:0.44]	0.13	0.99 [−1.77:2.17]	0.09
Physical Disability	1.00	**1.09** [**0.31**:**1.86**]	**0.00**	−0.07 [−1.15:1.00]	0.89
Psychological Disability	1.00	−0.47 [−1.64:0.68]	0.42	7.85 [−0.98:0.98]	1.00
Social Disability	1.00	−0.06 [−0.39:0.27]	0.71	^a^	^a^
Handicap	1.00	**0.02** [**0.01**:**0.39**]	**0.03**	0.02 [−0.19:0.24]	0.08

*Note:* Values in bold represent statistically significant differences (*p* ≤ 0.05). ^a^Collinearity.

**TABLE 5:** Mean (standard deviation) and the effect size (EF) in each domain of the OHIP‐Edent instrument.

OHIP‐Edent domains	1 Year	3 Years	5 Years	EF (5 × 1 years)	EF (5 × 3 years)
Functional Limitation	1.13 (1.30)	1.71 (1.76)	3.50 (1.79)	2.7	1.0
Pain	0.75 (1.19)	0.75 (1.39)	2.56 (2.25)	0.8	0.8
Psychological Discomfort	0.21 (0.66)	0.33 (1.01)	1.39 (1.20)	1.0	0.8
Physical Disability	0.38 (1.10)	0.46 (0.83)	1.22 (1.73)	0.5	0.4
Psychological Disability	0.17 (0.48)	0.17 (0.56)	0.61 (0.78)	0.5	0.5
Social Disability	0.13 (0.34)	0.00 (0.00)	0.06 (0.24)	0.2	0.2
Handicap	0.21 (0.83)	0.25 (0.74)	0.11 (0.32)	0.5	0.5
Global	2.96 (4.69)	3.63 (5.62)	9.44 (6.17)	1.0	0.9

**TABLE 6:** Multivariate regression analysis of the impact of chewing categorization on OHRQoL in the fifth year.

OHIP‐Edent domains	ST_X50			ST_B		
	Satisfactory (*n* = 11)	Unsatisfactory (*n* = 9)	*p*	Satisfactory (*n* = 10)	Unsatisfactory (*n* = 10)	*p*
	Ref.	Coef. [95% CI]		Ref.	Coef. [95% CI]	
Functional Limitation	1.00	1.76 [−0.08:3.61]	0.06	1.00	−0.69 [−2.50:1.10]	0.42
Pain	1.00	**2.50** [**0.25**:**4.74**]	**0.03**	1.00	−1.05 [−3.23:1.12]	0.31
Psychological Discomfort	1.00	0.73 [−0.58:2.05]	0.25	1.00	−0.57 [−1.86:0.70]	0.35
Physical Disability	1.00	−0.14 [−2.12:1.83]	0.87	1.00	−0.61 [−2.55:1.31]	0.50
Psychological Disability	1.00	**0.94** [**0.19**:**1.68**]	**0.01**	1.00	−0.20 [−0.92:0.52]	0.56
Social Disability	1.00	0.04 [−0.31:0.40]	0.79	1.00	−0.02 [−0.37:0.32]	0.86
Handicap	1.00	−0.11 [−0.46:0.22]	0.48	1.00	−0.18 [−0.52:0.15]	0.26
Global	1.00	5.61 [−0.81:12.05]	0.08	1.00	−3.42 [−9.70:2.84]	0.26

*Note:* Values in bold represent statistically significant differences (*p* ≤ 0.05).

## 4 Discussion

This clinical study is the first to systematically analyze the behavior of key outcome variables over a 5‐year period in IMO users rehabilitated with stud abutments. A robust analysis of the data revealed improvements in masticatory function and posterior bone remodeling, alongside declines in certain patient‐centered functional and physical outcomes, thereby rejecting the null hypothesis that no changes would occur in the monitored outcomes over 5 years. Despite overall improvements in masticatory function, 9 out of 20 IMO users did not achieve satisfactory ST_X50 values. This finding had a notable impact on specific OHIP domains, revealing that inadequate mastication adversely affected pain and psychological disability, underscoring the strong association between these factors.

The interpretation of the functional outcomes in this sample reveals a positive trend over 5 years, with a 7.5% reduction in the average particle size in ST_X50 between the first and fifth year, indicating improved food grinding over time. This finding aligns with previous studies that highlight the benefits of IMO (Sullivan and Feinn 2012; Naert et al. 2005; Zweers et al. 2015; Müller et al. 2015; Schuster et al. 2019; Possebon et al. 2020, 2021). A long‐term follow‐up study by Velasco‐Ortega et al. (2023) demonstrated that IMOs significantly contribute to more efficient and comfortable mastication, resulting in improved nutrition and quality of life. Furthermore, regarding the number of masticatory cycles and the time spent per cycle, there was a 10.56% increase in the number of cycles and a 6.32% reduction in cycle duration over the 5‐year period, compared to the first year. This suggests that patients rehabilitated with IMOs tend to chew more frequently and in less time as they adapt to the treatment. These results corroborate the findings of van der Bilt (2011), which emphasize the complexity of the relationship between the number of masticatory cycles, chewing time, and masticatory quality, influenced not only by biomechanical aspects but also by psychosocial factors.

Therefore, considering the results collectively, we can infer that prolonged use of IMOs is associated with improvements in both masticatory efficiency and quality. The reduction in ST_X50 particle size indicates more efficient food grinding, while the increased number of cycles reflects positive treatment adaptation. These results are also discussed by other authors (van der Bilt 2011), who indicate that patients improve their mastication in the long term due to better adaptation to this rehabilitation, with improvements possibly continuing up to 3 years after the installation of IMOs. These findings have significant implications for patients' oral and overall health, highlighting the role of this type of rehabilitation in promoting adequate masticatory function.

Beyond masticatory improvements, favorable changes in the posterior mandibular region were also observed. Contrary to earlier concerns that mucosa‐supported IMOs might contribute to posterior ridge resorption (Akeel et al. 1993; van der Bilt 2011), this study reported a 48.67% increase in bone area in the posterior region, indicating that IMOs are safe and capable of reversing significant posterior mandibular bone loss. This result is similar to that found by Kordatzis et al. (2003), who also reported bone preservation over a similar observation period. Patients with initially low mandibular bone volume, such as those in our sample, appear to benefit most from this type of rehabilitation (Reddy et al. 2002; Marcello‐Machado et al. 2017; Possebon et al. 2020). Furthermore, adaptation to IMOs over time appears to be influenced by a variety of factors, including prosthesis design, changes in supporting tissues, psychological adjustment, muscular adaptations, and neuromuscular control (Kimoto and Garrett 2003). These findings underscore the importance of long‐term follow‐up and monitoring to fully understand the functional dynamics of this treatment.

While early improvements in OHRQoL have been well documented in the early years (Faot et al. 2016; Marcello‐Machado et al. 2017; Miranda et al. 2019; Schuster et al. 2020), this study revealed a gradual decline in certain OHIP‐Edent domains over time. Although some studies report sustained benefits over five to ten years, our findings suggest that long‐term functional improvements may stabilize or even decline. Schuster et al. (2022) identified the need for adjustments in IMOs as a recurrent issue over time, negatively affecting quality of life. This reinforces the notion that such rehabilitations demand continuous maintenance—particularly regarding prosthesis retention, as highlighted in other studies (Alfadda et al. 2009; Cune et al. 2009; Martínez‐González et al. 2013). Although declines in functional limitation and physical disability domains, as well as the global score, were noted, the remaining 5 domains, which reflect social interaction and overall life satisfaction, remained relatively unaffected. This is particularly relevant given that unsatisfactory mastication has been associated with adverse outcomes, such as increased pain and psychological disability by the end of the fifth year. This finding emphasizes that masticatory quality is influenced not only by the biomechanical aspects of the rehabilitation but also by psychosocial determinants (Sutariya et al. 2021; Velasco‐Ortega et al. 2023).

Although this is a controlled longitudinal study with a robust 5‐year follow‐up, certain limitations should be acknowledged. The lack of a parallel control group receiving an alternative treatment, such as conventional complete dentures, limits direct comparison between interventions. Additionally, radiographic bone changes were assessed using two‐dimensional panoramic radiographs, which may be subject to dimensional distortion and limited precision in detecting subtle bone alterations, particularly in the posterior mandible. To mitigate this, standardized imaging protocols and consistent anatomical reference points were applied. While all participants were enrolled in a structured maintenance program, the impact of maintenance interventions on clinical and patient‐reported outcomes was not fully explored in this manuscript. Nonetheless, the controlled design, consistency of treatment protocol, and long‐term observation period significantly strengthen the validity and clinical relevance of the findings.

Finally, IMO‐based rehabilitation appears to be highly beneficial for edentulous patients, particularly those with limited mandibular bone volume. While functional declines in OHRQoL were observed, they did not significantly diminish overall treatment satisfaction. Regular follow‐up is essential to monitor prosthetic wear, retention loss, and other maintenance issues. Future research with extended follow‐up periods is necessary to further elucidate the long‐term performance and maintenance requirements of this rehabilitation approach.

## 5 Conclusion

Masticatory function and radiographic parameters of the posterior mandibular area in IMO wearers continue to change over the 5‐year period, with improvements in particle grinding indices and bone height gain in the posterior mandibular region, despite declines in certain functional outcomes related to oral health‐related quality of life. Additionally, unsatisfactory mastication negatively impacted domains such as pain and psychological disability, demonstrating the interrelationship between functional outcomes and patient‐centered outcomes.

## Author Contributions

**Anna Paula Da Rosa Possebon:** investigation, methodology, validation, visualization, formal analysis, writing – original draft. **Fernando Antônio Vargas Júnior:** investigation, writing – original draft, visualization. **Fernanda Isabel Román Ramos:** investigation, formal analysis, writing – original draft, validation, visualization, methodology. **Otacílio Luiz Chagas‐Júnior:** conceptualization, investigation, writing – review and editing, methodology, formal analysis, resources. **Luciana de Rezende Pinto:** resources, formal analysis, writing – review and editing, methodology, investigation. **Fernanda Faot:** conceptualization, investigation, funding acquisition, writing – review and editing, methodology, formal analysis, project administration, resources, supervision.

## Conflicts of Interest

The authors declare no conflicts of interest.

## Data Availability

The data that support the findings of this study are available on request from the corresponding author. The data are not publicly available due to privacy or ethical restrictions.
